# The inflammasome next door: characterizing pyroptosis induction in HCV-infected and uninfected bystander cells *in vitro*

**DOI:** 10.3389/fcimb.2025.1603739

**Published:** 2026-02-03

**Authors:** Hannah L. Wallace, Cassandra L. Gardner, Calvin N. Ezeanyaegbu, Jordan Wight, Andrew S. Lang, Rodney S. Russell

**Affiliations:** 1 Immunology and Infectious Diseases, Division of Biomedical Sciences, Faculty of Medicine, Memorial University, St John’s, NL, Canada; 2 Department of Biology, Faculty of Science, Memorial University, St John’s, NL, Canada

**Keywords:** hepatitis C virus (HCV), hepatocytes (hepatocyte-like cells), immune cells, inflammation, programmed cell death

## Abstract

Despite the fact that hepatitis C virus (HCV) can be cured with direct-acting antivirals in >95% of infected individuals, many of these individuals still have evidence of ongoing inflammation and some even go on to develop liver disease in the absence of ongoing infection. Previous work has demonstrated HCV-induced pyroptosis in both infected Huh-7.5 cells and uninfected bystander cells *in vitro*. Pyroptosis is an important form of inflammatory cell death that has been implicated in the pathogenesis of various viral infections. In HCV-infected cells, it has been unclear which step of the virus life cycle actually triggered pyroptosis. Using various virus constructs, we show here that fully infectious HCV production is required to trigger pyroptosis in infected Huh-7.5 cells. However, in S29 cells, which are 1000-fold less permissive to HCV infection, and express functional RIG-I, viral RNA replication was sufficient to trigger pyroptosis. In order to further understand bystander pyroptosis, we also investigated whether immune cells were influenced or triggered to undergo bystander pyroptosis in the context of HCV. We found that THP-1 cells (monocyte-like cells) undergo pyroptosis when co-cultured with HCV-infected Huh-7.5 cells both in their monocyte state as well as when differentiated into macrophages although this was not the case for Jurkat or Ramos cells. Together, our results help elucidate the trigger for pyroptosis in HCV-infected hepatocyte-like cells and further our understanding of how pyroptosis of infected hepatocytes can influence other cell types, particularly those that are inflammatory in nature, which may contribute to the pathogenesis of HCV.

## 1 Introduction

Pyroptosis is an inflammatory form of programmed cell death mediated by a protein complex dubbed the inflammasome. The inflammasome is formed when a trigger, such as a virus, or in some cases, part of a virus, activates a sensor such as nucleotide oligomerization domain, leucine-rich repeat, pyrin-domain containing protein 3 (NLRP3), absent in melanoma 2 (AIM2), or interferon gamma-inducible protein 16 (IFI16), among others. The activated sensor then oligomerizes with apoptosis-associated speck-like protein containing a CARD (caspase recruitment domain) (ASC), which then recruits caspase-1 to form the inflammasome complex. Caspase-1 then undergoes self-proteolysis to act as the effector enzyme. If the sensor contains a CARD such as nucleotide oligomerization domain, leucine-rich repeat, CARD containing 5 (NLRC5), the sensor is able to recruit caspase-1 directly without the requirement of ASC. Once the inflammasome is formed, caspase-1 cleaves gasdermin-D (GSDM-D), which forms pores in the cell membrane via trafficking of its N-terminus to the plasma membrane. Concurrently, caspase-1 also cleaves pro-IL-1 β and pro-IL-18 into their mature forms, which are subsequently released from cells via pores formed by GSDM-D. This process ultimately results in cell lysis that is associated with significant inflammation (extensively reviewed in Galluzzi et al., 2018).

Despite the availability of highly effective direct-acting antivirals (DAAs) which clear hepatitis C virus (HCV) in 95% of chronically infected individuals, there remain ~58 million people infected with HCV worldwide (World Health Organization, 2023). Global hepatitis elimination targets have been set by the WHO for 2030, although this has proven to be more difficult to achieve than anticipated. However, even if elimination goals are met, a small population of people will still go on to develop worsening liver disease, despite viral clearance (Welsch et al., 2017; Abe et al., 2020; D’Ambrosio et al., 2021; Montaldo et al., 2021). Inflammatory cytokines associated with inflammasome activation/pyroptosis are elevated in individuals living with chronic HCV infection (Falasca et al., 2006; Chattergoon et al., 2011; Veenhuis et al., 2016). A 2017 study by Burchill et al. assessed serum levels of a variety of inflammatory cytokines before and after DAA treatment. They reported that the levels of many inflammatory markers such as CXCL10, RIG-I, and IRF7 decreased in patient serum following cure with DAAs. Of particular interest is the additional finding that pyroptosis-associated cytokines IL-1 β and IL-18 did not decrease in serum following cure, suggesting that pyroptosis is still ongoing despite viral clearance (Burchill et al., 2017).

It has been shown that pyroptosis occurs in Huh-7.5 hepatocyte-like hepatoma cells infected with HCV as well as cells in the same cell culture that are uninfected (Kofahi et al., 2016). Kofahi et al. not only identified the phenomenon of pyroptosis during HCV infection, but also found that this phenomenon, unlike bystander apoptosis, was cell-contact independent. It was concluded that bystander pyroptosis is likely mediated by a soluble factor, but that factor remains unknown. Follow-up work identified significant cross-talk between the apoptosis and pyroptosis pathways during HCV infection, and showed that HCV utilizes cell death pathways as a mechanism of pathogenesis that aids in viral spread (Wallace et al., 2022).

Perhaps the most well-studied cell type in the context of inflammasome activation/pyroptosis are monocytes/macrophages. Indeed, for many years it was thought that only immune cells could undergo pyroptosis, and it was only recently that other cell types such as cardiomyocytes (Qiu et al., 2019), intestinal epithelia (Zhu et al., 2017; Tan et al., 2021), neurons (Han et al., 2020; Wan et al., 2020), airway epithelia (Deng et al., 2017), and, as we have shown, hepatocytes (Kofahi et al., 2016; Wallace et al., 2022) have also been documented to undergo pyroptosis both within and without the context of viral infections. Many studies have extensively detailed how monocytes/macrophages undergo pyroptosis and how this inflammatory process in these cells is thought to contribute to the pathology associated with numerous viral infections (Han et al., 2015; de Sousa et al., 2018; Platnich et al., 2018; de Castro-Jorge et al., 2019; Lê et al., 2019, Pan et al., 2019; Lien et al., 2021a; Pan et al., 2021; Rodrigues et al., 2021). As hepatocytes are not inherently inflammatory, most previous work on HCV-induced inflammasome activation/pyroptosis has focused on immune cells, with previous research relating to pyroptosis induction in the context of HCV reviewed by Wallace and Russell (Wallace and Russell, 2024). Previous work has documented that monocytes and macrophages exposed to HCV or HCV RNA undergo pyroptosis, although others have questioned whether merely exposure to HCV is sufficient to trigger inflammasome activation (Negash et al., 2013, 2019; Shrivastava et al., 2013; Chen et al., 2014; Wallace and Russell, 2024). Overall, this work aims to investigate and characterize HCV-induced pyroptosis and bystander pyroptosis in hepatocytes and immune cells.

## 2 Materials and methods

### 2.1 Cell culture

Huh-7.5 cells (gift from Apath, LLC) and S29 cells, a sub-clone of Huh7 cells that are 1000-fold less permissive to HCV due to decreased expression of CD81 (Russell et al., 2008; gift from Drs. S. Emerson and R. Purcell, National Institutes of Health, USA) were maintained at 37°C with 5% CO_2_ in complete medium (CM) containing Dulbecco’s Modified Eagle Medium (DMEM; with high glucose [4.5 g/L] and pyruvate; ThermoFisher Scientific, 11995073), supplemented with 10% heat-inactivated fetal bovine serum (FBS; ThermoFisher Scientific, 10438034) and 0.5% penicillin/streptomycin (Millipore Sigma, P4333-100ML).

THP-1 (gift from Dr. C. Moore, Memorial University), Ramos (gift from Dr. S. Christian via Dr. C. Moore, Memorial University), and Jurkat cells (gift from Dr. M. Grant, Memorial University) were maintained at 37°C with 5% CO_2_ in Immune Cell CM containing Roswell Park Memorial Institute 1640 media (RPMI; ThermoFisher, 11875119), supplemented with 10% heat-inactivated FBS, 1% penicillin/streptomycin, and 1X Antibiotic-Antimycotic Solution (Anti-Anti; 100X, Millipore Sigma, A5955).

#### 2.1.1 Co-culture of Huh-7.5 cells and immune cells

When Huh-7.5 cells were part of a co-culture, cells were seeded at a density of 1.5x10^5^ cells per well of a six-well plate (for flow cytometry) or 1x10^5^ cells per well of a 12-well plate (for ELISA). Huh-7.5 cells were infected with HCV 24 hours later. Following virus inoculation, on the same day, inoculum was replaced with immune cell CM and immune cells were added at a density of 2x10^5^ cells per well of a six-well plate or 7.5x10^4^ cells per well of a 12-well plate. Cells were then left for three or four-days post infection. No differences were noted when Huh-7.5 cells were cultured in Immune Cell CM (RPMI). Details regarding Huh-7.5 and THP-1 co-cultures for the purposes of microscopy specifically are detailed below in section 2.5.3.

**Figure 1:**
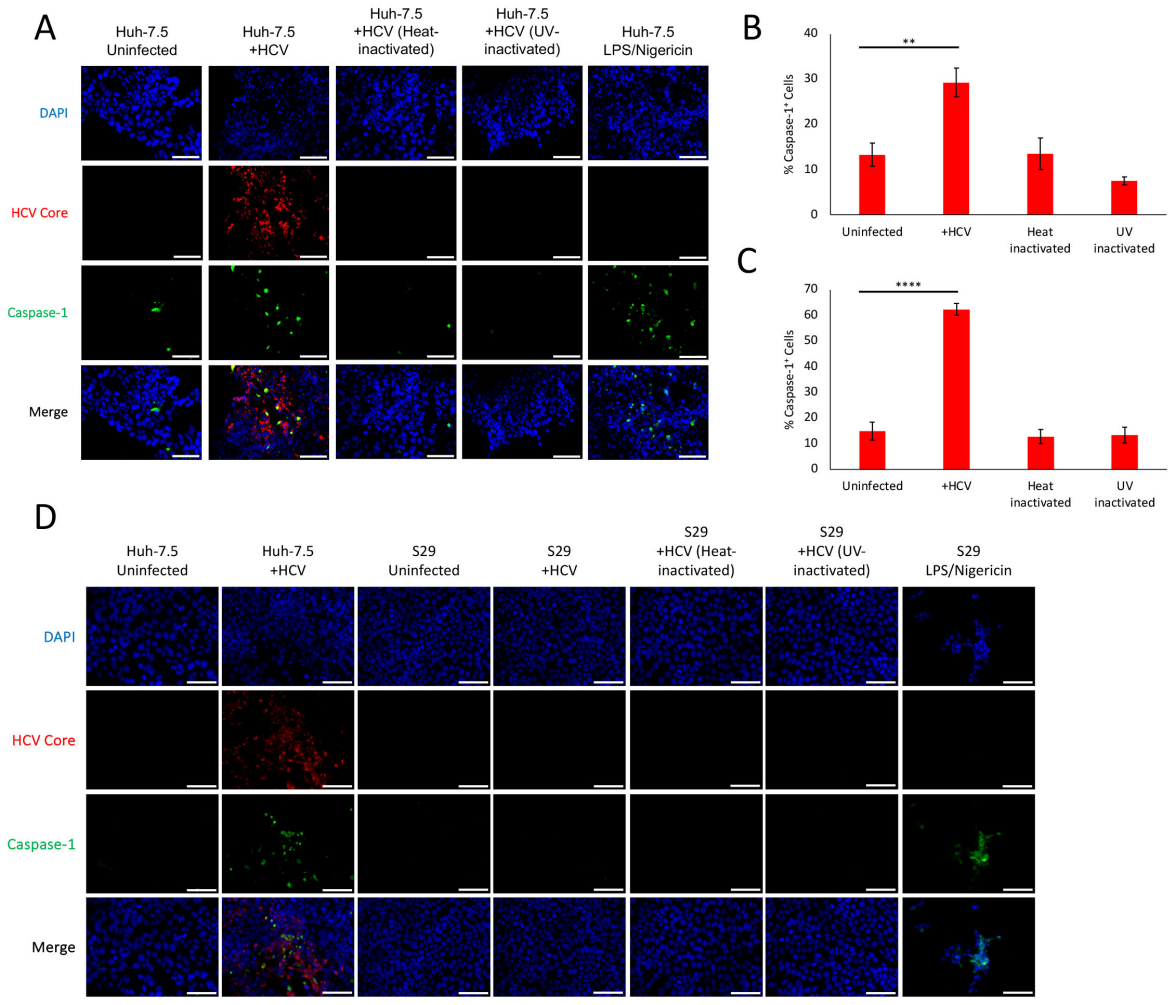
Viral entry alone is insufficient to trigger HCV-induced pyroptosis. Huh-7.5 cells were infected with HCV at MOI = 1 or left uninfected. Prior to infection, specific virus aliquots were either heat-inactivated or UV-inactivated. Twenty-four hours prior to staining, cells were treated with LPS/Nigericin as a positive control. **(A, D)** At 3 dpi cells were fixed using acetone and stained for cleaved caspase-1 (green), HCV core (red), and nuclei were stained with DAPI (blue). Analysis was performed using fluorescence microscopy. Scale bar, 100  μm. Data are representative of at least three independent experiments. **(B, C)** At 3 or 4 dpi cells were harvested and stained for cleaved caspase-1 before cells were fixed using the caspase-1 kit fixative. Cells were run on a CytoFLEX flow cytometer, and data were analyzed using Kaluza analysis software. Data are presented as the percent of total cells that were caspase-1^+^ with standard error. ** p< 0.005, **** p< 0.0001. Data are representative of at least two independent experiments performed in triplicate.

**Figure 2:**
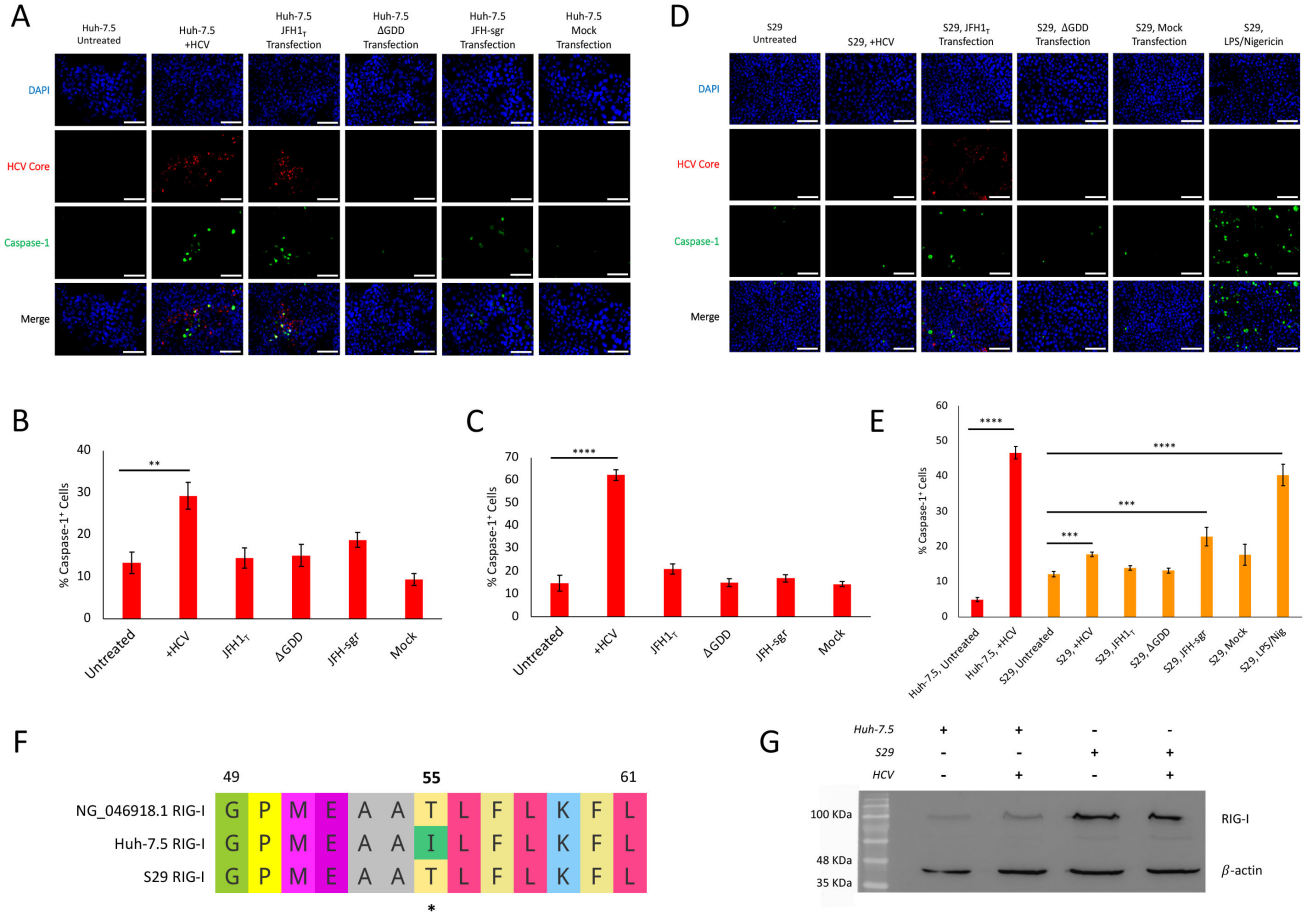
HCV-induced pyroptosis is dependent on fully infectious virion production in infected Huh-7.5 cells but not in untreated, bystander S29 cells. **(A–E)** Huh-7.5 or S29 cells were infected with HCV at MOI = 1, left untreated or transfected with RNA encoding for JFH1_T_, ΔGDD, JFH-sgr, or mock control. Twenty-four hours prior to staining, cells were treated with LPS/Nigericin as a positive control. **(A, D)** At 3 dpi cells were fixed using acetone and stained for cleaved caspase-1 (green), HCV core (red), and nuclei were stained with DAPI (blue). Analysis was performed using fluorescence microscopy. Scale bar, 100 μm. Data are representative of at least three independent experiments. **(B, C, E)** Huh-7.5 cells (red bars) and **(E)** S29 cells (yellow bars) were harvested and stained for cleaved caspase-1 prior to fixation, using the caspase-1 kit fixative at **(B)** 3 or **(C, E)** 4 dpi. **(B, C, E)** Cells were run on a CytoFLEX flow cytometer, and data were analyzed using Kaluza analysis software. Data are presented as the percent of total cells that were caspase-1^+^ with standard error. ** p<0.005, *** p<0.0005, **** p<0.0001. **(F)** Multiple sequence alignment highlighting the T55I substitution in the RIG-I gene of Huh-7.5 cells that is not in the S29 cells or the human reference sequence (NG_046918.1). **(G)** Western blotting of cell lysates from Huh-7.5 and S29 cells, with or without HCV infection. Membranes were probed for RIG-I and β-Actin. **(A–E, G)** Data are representative of at least three independent experiments or two independent experiments performed in triplicate.

### 2.2 Virus stocks

A cell culture-adapted strain of HCV, known as JFH1_T_ (Russell et al., 2008; Jones et al., 2011), was used for this study. To generate virus stocks, 1x10^6^ Huh-7.5 cells were seeded in 10-cm culture dishes. Approximately 24 hours later, cells were inoculated at a multiplicity of infection (MOI) of 1 and incubated for 3 hours, after which inoculum was replaced with fresh CM. Culture fluids were harvested four days post-infection (dpi) and virus titre was determined using a limiting dilution focus-forming assay which has been described previously (Wallace et al., 2022).

#### 2.2.1 Heat inactivated HCV

HCV was heat-inactivated by heating 1 mL of HCV stock in a 1.5-mL tube in a heat block at 92°C for 30 minutes, a protocol adapted and verified for use for SARS-CoV-2 inactivation (Pastorino et al., 2020).

**Figure 3:**
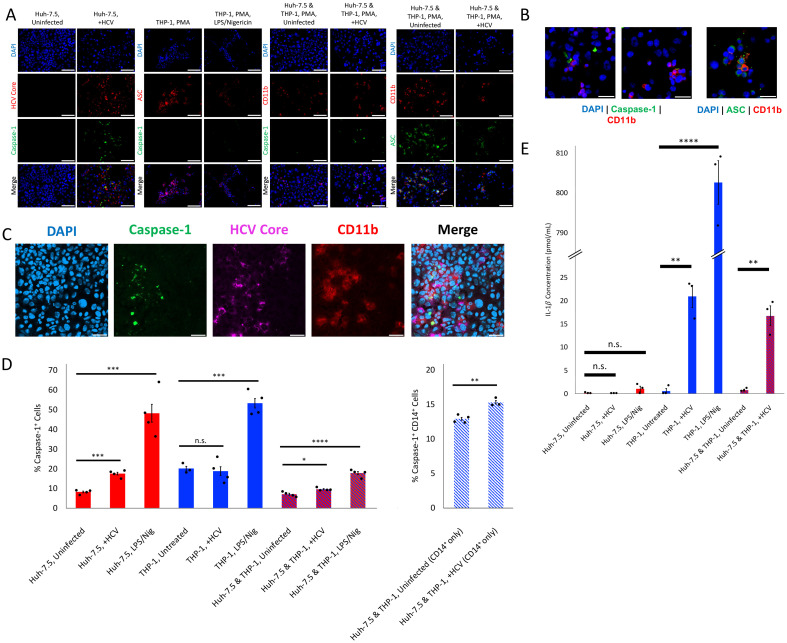
Bystander pyroptosis occurs in THP-1 cells co-cultured with HCV-infected Huh-7.5 cells. Huh-7.5 cells were plated and one day later, infected with HCV at MOI = 1. Immediately following virus inoculation, THP-1 cells were added to co-cultures or individual mono-cultures. Co-cultures contained fewer cells of each cell type than a mono-culture alone, as experiments were performed within the same volume and area. **(A–C)** At 1 dpi, THP-1 monocultures or co-cultures were treated with PMA, to differentiate the cells into adherent macrophages. At 2 dpi, culture fluids were replaced with fresh CM. At 4 dpi, cells were fixed with acetone and stained for cleaved caspase-1 or ASC (green), CD11b or CD14 (red), and for some experiments, HCV core (pink) and nuclei were stained with DAPI (blue). Analysis was performed using fluorescence microscopy. **(A, B)** Scale bar, 100 μm. **(B)** Examples of bystander pyroptosis of THP-1 cells, with white arrows indicating THP-1 cells undergoing pyroptosis. **(C)** Scale bar, 50 μm. **(A–C)** Data are representative of at least three independent experiments. **(D)** At 4 dpi, cells were harvested and stained for cleaved caspase-1 before being fixed with the caspase-1 kit fixative. Then cells were stained for CD14. Cells were run on a CytoFLEX flow cytometer, and data were analyzed using Kaluza analysis software. Data are presented as the mean percent of total cells that were caspase-1^+^ with standard error. Data are representative of at least three independent experiments. **(E)** For the IL-1 β ELISA experiments, cell culture fluids were collected at 3 dpi, and clarified by centrifugation. Culture fluids were then used to analyze concentration of IL-1 β released from cells using a SpectraMax Mini microplate reader. Results are presented as the concentration of IL-1 β in pmol/mL and are shown along with standard error. Data are representative of at least two independent experiments performed in triplicate. **(D, E)** Huh-7.5 cells (red bars), THP-1 cells (blue bars), Huh-7.5 and THP-1 co-culture (red bars with blue hatching), **(D)** CD14^+^ cells from Huh-7.5 and THP-1 co-culture (white bars with blue hatching). **(D, E)** *p<0.05; **p<0.005; ***p<0.0005; **** p<0.0001.

#### 2.2.2 UV-inactivated HCV

HCV was inactivated using a UV Stratalinker 1800 (Stratagene) by dosing 500 μL of HCV stocks, in a 35mm plate (without the cover) with 120,000 μJ of UV, 7 times with 2 minute intervals between each dose (adapted from Amaya et al., 2014).

### 2.3 *In vitro* transcription and RNA transfection

Previously synthesized DNA plasmids encoding various RNA constructs were linearized by *Xba*l restriction digest (*Xba*l GQ, Promega, R6185) and *in vitro* transcribed using the T7 RiboMAX™ Express large scale RNA production system (Promega, P1320) according to manufacturer instructions. RNA encoding either JFH1_T_ or JFH1_T_ ΔGDD (non-replicating RNA; referred to as *Δ*GDD) or JFH-sgr (sub-genomic replicon, developed by Lohmann et al., 1999) was transfected into cells using DMRIE-C (Thermofisher, 10459-014) as per manufacturer instructions. The RNA encoding JFH1_T_ results in production of infectious virions. The ΔGDD JFH1_T_ RNA harbours a three amino acid deletion in the active site of the NS5B polymerase, rendering it incapable of undergoing viral replication as previously described (Jones et al., 2011). The JFH-sgr RNA encodes only the non-structural proteins of HCV and, therefore, does not generate progeny virions, but does undergo viral RNA replication.

**Figure 4:**
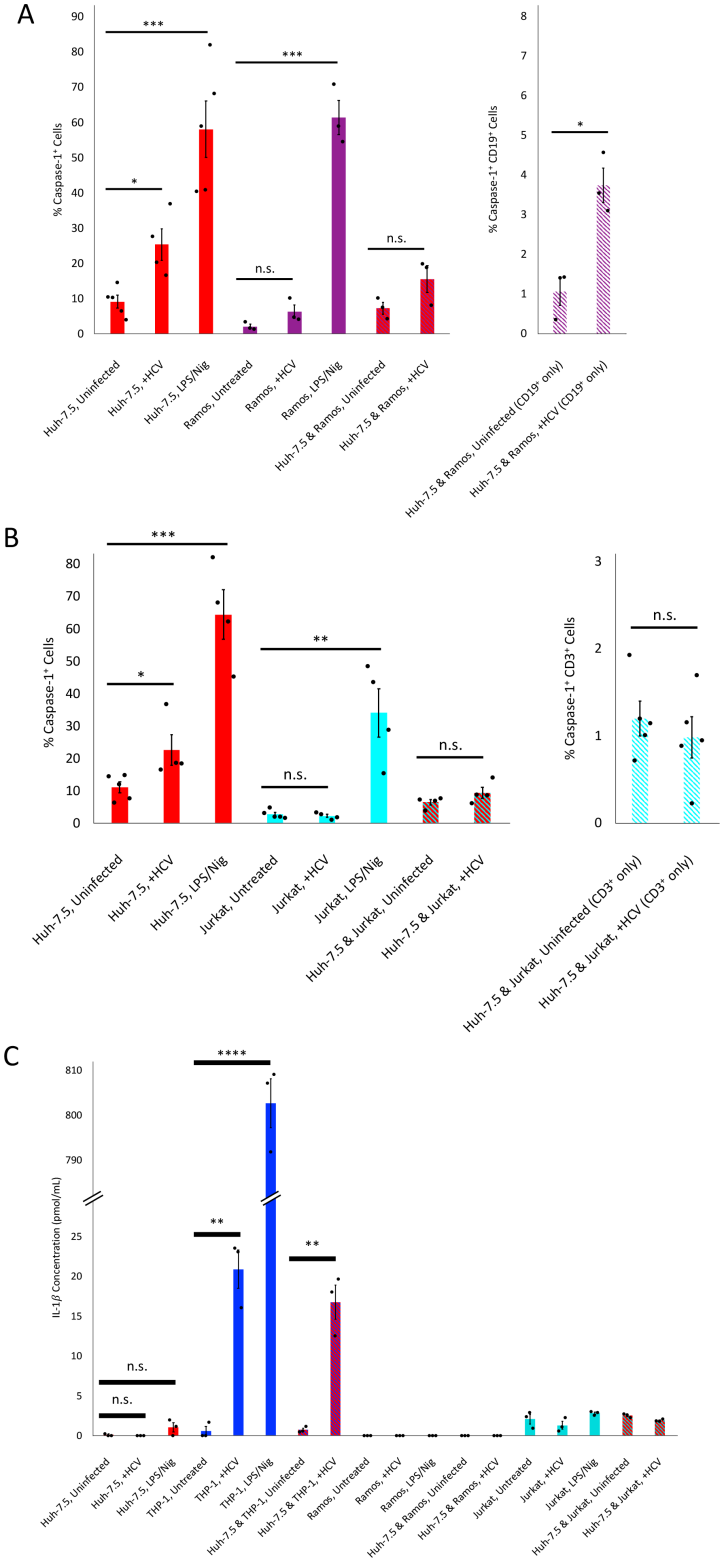
Ramos and Jurkat cell lines are affected differently by HCV-induced bystander pyroptosis. Huh-7.5 cells were infected with HCV at MOI = 1 or left uninfected. Following virus inoculation, Ramos or Jurkat cells were added to the respective co-culture conditions. Ramos and Jurkat cells were also inoculated with HCV at an MOI = 1. **(A, B)** For flow cytometry experiments, cells were left for 4 days following infection. At 4 dpi, cells were collected and stained for cleaved caspase-1 using a specific probe and fixed using fixative from the caspase-1 probe kit. Cells were subsequently stained with antibodies against CD19 or CD3, for Ramos or Jurkat cells, respectively. Cells were then run on a CytoFLEX flow cytometer and data were analyzed using Kaluza analysis software. Results are presented as mean percentage of caspase-1^+^ cells and are shown with standard error. Data are representative of at least three independent experiments. **(C)** For the IL-1 β ELISA experiments, cells were left for 3 days, before cell culture fluids were collected, and clarified by centrifugation. Culture fluids were then used to quantify IL-1 β release from cells. The results presented in **Figure 3E** for THP-1 cells are also included here for comparison purpose. Results are presented as the concentration of IL-1 β in pmol/mL and are shown along with standard error. **(A–C)** Huh-7.5 cells (red bars). **(A, C)** Ramos cells (purple bars), Huh-7.5 and Ramos co-culture (red bars with purple hatching). **(A)** CD19^+^ cells from Huh-7.5 and Ramos co-culture (white bars with purple hatching). **(B, C)** Jurkat cells (light blue), Huh-7.5 and Jurkat co-culture (red bars with light blue hatching). **(B)** CD3^+^ cells from Huh-7.5 and Jurkat co-culture. **(C)** THP-1 cells (blue bars), Huh-7.5 and THP-1 co-culture (red bars with blue hatching). Data are representative of at least two independent experiments performed in triplicate. **(A–C)** *p<0.05; **p<0.005; ***p<0.0005; ****p<0.0001.

### 2.4 Pyroptosis induction by lipopolysaccharide/Nigericin treatment

To induce pyroptosis as a positive control in Huh-7.5 or S29 cells, lipopolysaccharide from *Escherichia coli* 0127:B8 (LPS; *in vitro* LPS, Millipore-Sigma, L5024-10MG) was added to cells at 5 μg/mL and incubated for 3 hours at 37°C. Following incubation with LPS, Nigericin (sodium salt, InvivoGen, tlrl-nig) was added at a concentration of 16.8 μM and left overnight at 37°C. To induce pyroptosis as a positive control in immune cells, LPS was added to cells at 3 μg/mL and incubated for 3 hours at 37°C. Following incubation, Nigericin was added at a concentration of 11.0 μM and left overnight at 37°C. This treatment was always performed the day prior to staining/cell harvest.

### 2.5 Fluorescence microscopy

#### 2.5.1 Monoculture of Huh-7.5 or S29 cells

When either Huh-7.5 or S29 cells were used alone, cells were seeded at a density of 1x10^5^ cells per well in 2-well chamber slides (Fisher Scientific, 12-565-16). The following day (0 dpi), cells were infected with HCV at an MOI = 1, transfected with RNA, or left uninfected/untreated. Cells were allowed to propagate for three or four days post infection/transfection (3/4 dpi).

#### 2.5.2 Monoculture of THP-1 cells

When THP-1 cells were used alone, cells were seeded at a density of 2.5x10^5^ cells per well in 2-well chamber slides (Fisher Scientific, 12-565-16). The day following plating, cells were treated with phorbol-12-myristate-13-acetate (PMA; Millipore-Sigma, 5005820001) at a 1:100,000 dilution and allowed to differentiate for 24 hours. Once the cells were differentiated and attached to the bottom of the well, media was changed and cells were left for an additional 24 hours prior to further manipulation.

#### 2.5.3 Co-culture of Huh-7.5 and THP-1 cells

When Huh-7.5 cells were part of a co-culture, the cells were seeded at a density of 7.5x10^4^ cells per well. Huh-7.5 cells were infected with HCV 24 hours later. Following virus inoculation, on the same day, inoculum was replaced with immune cell CM and THP-1 cells were added at a density of 1.5x10^5^ cells per well. Therefore, the proportion of each cell type in the co-culture is different from that of the individual mono-cultures. For conditions including THP-1 cells, the day after their addition to the culture (1 dpi), cells were treated with PMA at a 1:100,000 dilution and allowed to differentiate for 24 hours. Once the cells were differentiated and attached to the bottom of the well, media was changed and cells were left for an additional 24 hours prior to further manipulation.

#### 2.5.4 General staining

For all cell conditions, caspase-1 was visualised using a FAM-FLICA^®^ caspase-1 inhibitor kit (ImmunoChemistry Technologies, 98), referred to as a caspase-1 probe (Compan et al., 2015; Boucher et al., 2018; Guo et al., 2018; Baroja-Mazo et al., 2019; Gaul et al., 2021). The staining protocol used in this study, including the use of the caspase-1 probe, is extensively detailed in Wallace et al., 2022. Briefly, on 3 or 4 dpi, CM was removed from cells and replaced with 30X FAM-FLICA^®^ caspase-1 probe in CM and incubated with the cells for 2 hours prior to washing, fixing, and subsequent antibody staining. We have previously demonstrated that increases in the detection of cleaved caspase-1, using this probe, either by fluorescence microscopy or flow cytometry, is indicative of pyroptosis in our system, including subsequent downstream cleavage of GSDM-D and LDH release (Kofahi et al., 2016; Wallace et al., 2022). Antibody staining following caspase-1 probe staining, included primary antibodies against HCV core protein (Anogen, product MO-I40015B), CD11b (Abcam, M1/70, ab8878), or ASC (Adipogen, AL177, AG-25B-0006-C100). These antibodies were used at dilutions of 1:200, 1:200, and 1:100, respectively, in solutions of 5% bovine serum albumin (BSA; Millipore Sigma, A9418-50G) in PBS. If the anti-HCV core antibody was used alone, slides were incubated with the antibody solution for 20 minutes at room temperature. If the anti-CD11b or anti-ASC antibodies were used, with or without the anti-HCV core antibody, slides were incubated overnight at 4°C in humid surroundings. Following incubation, regardless of timing, slides were washed in PBS for 5 minutes prior to a 20-minute incubation with secondary antibodies at room temperature. Secondary antibodies included goat anti-mouse Alexa Fluor^®^ 594 or goat anti-mouse Alexa Fluor^®^ 647 (used with the anti-HCV Core antibody; Thermofisher, A11020 or A32728, respectively), goat anti-rabbit Alexa Fluor^®^ 488 (used with the anti-ASC antibody; Thermofisher, A11008), and goat anti-rat Alexa Fluor^®^ Plus 594 (used with the anti-CD11b antibody; Thermofisher, A48264). All secondary antibodies were used at 1:100 dilution in PBS. An additional 5-minute wash in PBS was performed following secondary antibody staining for all conditions. Slides were mounted using VECTASHIELD Vibrance Mounting Medium with DAPI (BioLynx, VECTH180010).

#### 2.5.5 Staining for calnexin with or without LipidTOX™

If the anti-calnexin antibody and/or LipidTOX™ were used, cells were first stained with the caspase-1 probe, as described above, then washed with 300 μL of PBS directly in the slide wells. Then cells were fixed with 4% paraformaldehyde (PFA; 400 μL) by incubating at room temperature for 15 minutes. Cells were then rinsed with PBS (300 μL). Cells were permeabilized with 0.5% Triton X-100 and covered with a coverslip, and incubated at room temperature for 30 minutes. Slides were then rinsed in PBS and allowed to dry prior to staining. The anti-calnexin antibody (Abcam, ab10286) and the anti-HCV core antibody (same as above) were both used at 1:200 dilution in 5% BSA in PBS and incubated overnight at 4°C in humid surroundings. The secondary antibody staining was the same as above, using goat anti-rabbit Alexa Fluor^®^ Plus 594 (Thermofisher, A11037) for the anti-calnexin antibody, and either goat anti-mouse Alexa Fluor^®^ 594 (Thermofisher, A11020) or goat anti-mouse Pacific Blue™ (Thermofisher, P10993) for the anti-HCV core antibody. Once antibody staining was completed, a 1:200 dilution of LipidTOX™ 647 (LipidTOX™ deep red neutral lipid stain, ThermoFisher, H34477) in VECTASHIELD mounting media (BioLynx, VECTH170010) was prepared and slides were mounted as described above.

Slides were visualized using a Zeiss Axio Imager.M2 immunofluorescence microscope, an Olympus Fluoview FV1000 laser scanning microscope, or a Zeiss LSM 900 with Airyscan microscope. All microscopy images within this study are representative of at least three independent experiments.

### 2.6 Flow cytometry

#### 2.6.1 Quantification of caspase-1^+^ Huh-7.5 or S29 cells

Flow cytometry was used to quantify the proportion of caspase-1^+^ cells using the same FAM-FLICA^®^ Caspase-1 Assay Kit as above. Flow cytometry analysis was performed using a detailed and previously published protocol (Wallace et al., 2022). Analysis was performed as previously published using Kaluza software (version 2.1.1; Beckman Coulter; Wallace et al., 2022). The gating strategy for this analysis is provided in **Supplementary Figure 1**.

#### 2.6.2 Quantification of caspase-1^+^ immune cells

When immune cells were used in flow cytometry experiments they were stained for cleaved caspase-1 as per Wallace et al., 2022 and then stained for various CD markers conjugated with a Brilliant Violet 421™ fluorophore using antibodies against CD19 (BioLegend, 363017/363018), CD3 (BioLegend, 317343/317344), or CD14 (BioLegend, 301829/301830) for B cells, T cells, and monocytes, respectively. Following fixation (performed as per Wallace et al., 2022), cells were washed with 500 μL of 3% BSA in PBS, then resuspended in 100 μL of antibody solution (including 5 μL of antibody in PBS; ~5 μL per 10^6^ cells) and incubated at 4°C for 30 minutes. Cells were then washed a final time with 1% BSA in PBS before their final resuspension in 500 μL of 1% BSA for analysis. All flow cytometry experiments were performed as at least three independent experiments, or alternatively, repeated at least twice in triplicate. Analysis was performed using Kaluza software (version 2.1.1; Beckman Coulter) where gates were set using controls of each cell line individually and of the co-culture that included unstained controls, fluorescence minus 1 controls, and positive controls of heat-shocked cells as well as LPS/Nigericin treatment. The gating strategy used for this analysis can be found in **Supplementary Figure 2**.

### 2.7 RNA extraction, PCR amplification, and sequencing of the RIG-I transcript

Huh-7.5 and S29 cells were trypsinized, pelleted, and resuspended in PBS. RNA was exacted from approximately 1.5 million cells using the RNeasy Plus Mini Kit (Qiagen, 74134) as per the manufacturer’s instructions.

Specific PCR primers were designed to amplify multiple overlapping fragments covering the entire coding sequence of human RIG-I (**Supplementary Table 1**). RT-PCR was performed in 25 μL reactions using the OneTaq^®^ One-step RT-PCR Kit (New England Biolabs, E5315S) as followed: 12.5 μL of 2X OneTaq One-step Reaction Mix, 1 μL of 2X OneTaq One-step Enzyme Mix, 1 μL of each forward and reverse primer (10 μM), 8 μL nuclease-free water, and 1.5 μL of RNA. Cycling parameters for all amplicons were as followed: 48°C for 30 min, 95°C for 5 min, 40 cycles of 94°C for 15 sec, 50°C for 30 sec, and 68°C for 75 sec, followed by a final extension at 68°C for 7 min. PCR products were subjected to electrophoresis for visualization, and amplicons were purified using the Monarch^®^ Spin PCR & DNA Cleanup Kit (New England Biolabs, T1130L) and subjected to Sanger sequencing at The Hospital for Sick Children (Toronto, Canada). Sequences were mapped to the human RIG-I reference sequence (GenBank: NG_046918.1) and translated into the open reading frame using Geneious 11.1.5 (Dotmatics). The RIG-I complete coding sequences for Huh-7.5 and S29s have been deposited in GenBank under accession numbers PX629822 and PX629823, respectively.

### 2.8 Western blotting

Western blotting was performed as described previously (Wallace et al., 2022). Briefly, cells were seeded in 6-well plates and infected with HCV at MOI = 1 or left uninfected the following day. At 3 dpi, cells were harvested and protein extracted. Protein samples were separated by SDS-PAGE and transferred to nitrocellulose membranes (Amersham/Cytiva, 10600065). Membranes were incubated with antibodies recognizing RIG-I (Alme-1, Adipogen, AG-20B-0009) and β-Actin (C4, Santa Cruz Biotechnology, Inc., sc-47778). Signal was detected using Cytiva Amersham™ ECL Select™ Western Blotting Detection Reagent (FisherScientific, 45000999).

### 2.9 IL-1 β ELISA

ELISAs to detect and quantify released, extracellular IL-1 β were performed as per manufacturer’s instructions (Human IL-1 β ELISA Kit, Invitrogen, BMS224-2) using a 4-parameter curve fit. Cell culture fluids were collected at 3 dpi, clarified by centrifugation and frozen at -80°C for storage until analysis. Experiments were performed in triplicate, at least twice, and analyzed using a SpectraMax Mini microplate reader (Molecular Devices).

### 2.10 Statistical analyses

Statistical analyses for flow cytometry and ELISA data were performed using the Analysis ToolPak in Microsoft Excel (2024). For both flow cytometry and ELISA data, one-way ANOVA was used to compare conditions, where p-values< 0.05 were considered statistically significant. All flow cytometry and ELISA statistical analyses were evaluated using at least three independent experiments or two independent experiments performed in triplicate.

### 2.11 Data visualization

Flow cytometry and ELISA data were visualized using Microsoft Excel (2024). The multiple sequence alignment for RIG-I at residue 55 and surrounding region was visualized in R (v4.5.0; R Core Team, 2021) using ggmsa (v1.16.0; Zhou et al., 2022).

### 2.12 RT-qPCR for detection of Viral RNA

RT-qPCR was performed as per (Oraby et al., 2021). Briefly, Huh-7.5 and S29 cells were transfected with JFH-sgr or left untreated. At one hour, one day, two days, three days, and four days following transfection, cells were harvested and RNA isolated. RNA was subjected to RT-qPCR to detect HCV NS5B RNA.

## 3 Results

### 3.1 Viral entry alone is insufficient to trigger HCV-induced pyroptosis

Previously published work demonstrated that HCV induces pyroptosis of infected and uninfected Huh-7.5 cells, the latter of which was shown to be contact-independent (Kofahi et al., 2016; Wallace et al., 2022). Bystander pyroptosis was visualized (**Supplementary Figure 1**) and the mechanism(s) responsible for bystander pyroptosis was explored. To determine if entry and/or interactions of the virus with cell-surface receptors was sufficient to trigger pyroptosis in the absence of an infectious virus life cycle, HCV stocks were heat- or UV-inactivated prior to addition to cell cultures. Huh-7.5 cells were infected with HCV, heat-inactivated HCV, UV-inactivated HCV, left uninfected or treated with LPS/Nigericin as a positive control. Cells were then left to grow for 3 or 4 days prior to staining, using a probe for active caspase-1 (indicative of pyroptosis) and antibodies against HCV core protein. Cells were visualized via fluorescence microscopy and increased levels of caspase-1 were observed in cells infected with HCV compared to cells left uninfected (**Figure 1A**). This is accompanied by many caspase-1 positive cells that did not appear to be infected, indicating bystander pyroptosis. To quantify the percentage of cells that were caspase-1^+^, cells were harvested and stained using the caspase-1 probe and analyzed by flow cytometry. As previously reported (Kofahi et al., 2016; Wallace et al., 2022), HCV infection resulted in significantly more caspase-1^+^ cells when compared to cells that were uninfected at either 3 or 4dpi (29.3% (SEM ±3.23) of HCV-infected cells compared to 13.3% (SEM ±2.53) of uninfected, p<0.005, and 62.3% (SEM ±2.3) compared to 14.9% (SEM ±3.48), p<0.0001, respectively; **Figures 1B, C**). Neither the heat-inactivated nor UV-inactivated HCV were able to productively infect cells (**Figure 1A**) or induce caspase-1 levels higher or significantly different from that of uninfected cells (**Figures 1A–C**). To further confirm these findings, S29 cells were also inoculated with HCV, heat-inactivated HCV, UV-inactivated HCV, left uninfected or treated with LPS/Nigericin as a positive control. S29 cells are a sub-clone of Huh7 cells and are ~1000-fold less permissive to HCV infection due to extremely low levels of CD81 expression (Russell et al., 2008), thereby essentially eliminating the possibility of low-level viral replication having an impact on our results. None of the S29 conditions displayed increased active caspase-1 when compared to uninfected cells except for the cells treated with LPS/Nigericin as a positive control (**Figure 1D**). Taken together, these results indicate that extracellular interactions of HCV with hepatocyte-like cells are insufficient to trigger pyroptosis. These results also eliminate these interactions as potential mechanisms of bystander pyroptosis induction of uninfected cells.

### 3.2 HCV-induced pyroptosis of Huh-7.5 cells is dependent on fully infectious virion production

To determine which step of the viral lifecycle is responsible for pyroptosis induction by HCV, Huh-7.5 cells were infected with HCV, transfected with JFH1_T_, ΔGDD, JFH-sgr, mock transfected, or left untreated. Cells were then left to grow for 3 or 4 days prior to staining for active caspase-1 and HCV core protein. As demonstrated previously, HCV infection or JFH1_T_ RNA transfection resulted in an increase of caspase-1 activation with the majority of the cells infected (**Figures 2A****;****1A, D**; Kofahi et al., 2016; Wallace et al., 2022). None of the other conditions were sufficient to induce increased levels of caspase-1 activation (**Figure 2A**). To further confirm these findings, the experiment was repeated but cells were subjected to flow cytometry to quantify the percentage of caspase-1^+^ cells present in each condition at 3 or 4 dpi. On the given day, cells were collected, stained using the caspase-1 probe, and analyzed by flow cytometry. A significantly higher percentage of cells infected with HCV, 29.28% (SEM ±3.23) and 62.4% (SEM ±2.3), were caspase-1^+^ at both 3 and 4 dpi, respectively, in comparison to uninfected cells, 13.3% (SEM ±2.53) and 14.9% (SEM ±3.48), p<0.005 and p<0.0001 at 3 and 4 dpi, respectively (**Figures 2B, C**). To further elucidate which step of the HCV life cycle is involved with the triggering of pyroptosis, we performed the same experiments again employing S29 cells to remove multiple rounds of infectious virion production as a factor. As expected, transfection of S29 cells with JFH1_T_ RNA resulted in increased levels of caspase-1 compared to untreated S29 cells, since S29 cells can produce progeny virions when transfected with the positive-sense HCV RNA but additional cells cannot be infected (**Figure 2D**). We then compared the percentage of caspase-1^+^ cells by flow cytometry and found that S29 cells stimulated with LPS and Nigericin led to the activation of caspase-1 in a significantly higher proportion of the cells (40.36%, SEM ±3.06) when compared to the untreated S29 cells (12.15%, SEM ±0.75), confirming that S29 cells are capable of classical inflammasome activation (p<0.0001). Unexpectedly, we found significantly increased numbers of caspase-1^+^ S29 cells in cultures inoculated with infectious HCV (17.84%, SEM ±0.64) when compared to the uninfected S29 cells (12.15%, SEM ±0.75; p<0.0005; **Figure 2E**). However, HCV infections are done using stocks of HCV, meaning harvested cell culture fluids, which by nature, includes any soluble factors released from the cells from which the culture fluids were harvested. Interestingly, we found increased numbers of caspase-1^+^ S29 cells induced by transfection with JFH-sgr (22.85%, SEM ±2.61) compared to untreated S29 cells (12.15%, SEM ±0.75; p<0.005; **Figure 2E**) although we did not see the same effect when Huh-7.5 cells were used (**Figure 2B, C**). Together, these data clearly demonstrate that the fully infectious HCV life cycle is necessary for pyroptosis induction in infected Huh-7.5 cells. However, this was not the case for S29 cells, where exposure to HCV or transfection with the JFH-sgr were both sufficient to induce pyroptosis. To determine whether this discrepancy was due to differences in RNA replication between the two cell lines, Huh-7.5 and S29 cells were transfected with the JFH-sgr, RNA collected and subjected to RT-PCR for detection of viral NS5B at one hour, and one, two, three, and four days post-transfection. As the average C_T_ values differed by less than 3.32, which represent a 1 log change in viral RNA load, levels of viral RNA between Huh-7.5 and S29 across each of the sampled timepoints are functionally equivalent. This indicates there is no difference in RNA production between the two cell lines (**Supplementary Table 2**). Instead, the differences between the two cell lines are likely due to the fact that S29 cells, like parental Huh7 cells and the human RIG-I reference sequence, encode a threonine at position 55 of RIG-I, allowing for RIG-I to function normally (**Figure 2F**). This is in contrast to Huh-7.5 cells, which have an isoleucine at position 55, which previously studies have shown results in non-functional RIG-I (**Figure 2F**; Sumpter et al., 2005). Additionally, Huh-7.5 cells appeared to have less RIG-I expression compared to S29 cells (**Figure 2G**), further supporting that this difference between the two cell lines may explain the differences in pyroptosis induction.

### 3.3 Bystander pyroptosis of THP-1 cells co-cultured with HCV-infected hepatocytes

It has been previously documented that the levels of inflammatory cytokines IL-1 β and IL-18 are significantly elevated in the serum of individuals living with HCV (Falasca et al., 2006; Chattergoon et al., 2011; Veenhuis et al., 2016; Burchill et al., 2017; Welsch et al., 2017; Abe et al., 2020; D’Ambrosio et al., 2021; Montaldo et al., 2021). Given the fact that chronic HCV infection is associated with inflammation, and the understanding that hepatocytes are not known to be particularly inflammatory in nature, we investigated whether bystander pyroptosis would also be observed in immune cells co-cultured with HCV-infected hepatocyte-like cells. Since it is well documented that monocytes and macrophages can undergo pyroptosis accompanied by substantial inflammatory cytokine release, we performed an experiment in which Huh-7.5 cells were co-cultured with THP-1 cells (a monocyte-like cell line that can be differentiated into a macrophage-like phenotype). We aimed to further investigate bystander pyroptosis and to determine whether THP-1 cells are susceptible to bystander pyroptosis. For co-culture infections, we inoculated Huh-7.5 cells with HCV or left them uninfected before adding THP-1 cells. Even though THP-1 cells cannot be infected with our HCV isolate, JFH1_T_ (Sarhan et al., 2012), our experiments (**Figure 2**) showed that exposure to HCV can trigger pyroptosis of uninfected cells. Therefore, we included a condition in these experiments whereby we exposed the THP-1 cells to HCV. To visualize bystander pyroptosis of THP-1 cells, we differentiated the THP-1 cells into adherent macrophage-like phenotypes using PMA. Cells were stained for caspase-1 or ASC, HCV core protein, and/or CD11b (as a marker of the THP-1 cells) and nuclei were stained using DAPI (**Figures 3A–C**). Some cells that are CD11b^+^ (the THP-1 cells) also showed caspase-1 activation or ASC speck formation (**Figures 3A–C**). We also quantified the amount of caspase-1^+^ cells in both HCV-infected and uninfected mono- and co-cultures of Huh-7.5 and THP-1 cells by flow cytometry. As expected, there was a significantly higher percentage of caspase-1^+^ Huh-7.5 cells in samples that were infected with HCV than there was in uninfected Huh-7.5 cells (17.48% (SEM ±0.69) compared to 8.17% (SEM ±0.43); p<0.0005; **Figure 3D**). The overall percentage of caspase-1^+^ cells was significantly higher in the co-culture of HCV-infected Huh-7.5 and THP-1 cells than it was in the uninfected co-culture condition (9.57% (SEM ±0.3) compared to 7.07% (SEM ±0.47); p<0.05; **Figure 3D**). When the co-cultures were separated based on CD14^+^ cells (to examine the THP-1 cells specifically), although the overall percentage was low, there were significantly more caspase-1^+^ THP-1 cells when they were co-cultured with HCV-infected Huh-7.5 cells than when co-cultured with uninfected Huh-7.5 cells (15.33% (SEM ±0.24) compared to 12.93% (SEM ±0.21); p<0.005; **Figure 3D**). The same experimental conditions were used to quantify IL-1 β secretion by ELISA. These data revealed a significant increase in the amount of IL-1 β secreted by THP-1 cells exposed to HCV compared to unexposed cells (0.57 pmol/mL (SEM ±0.57) compared to 20.88 pmol/mL (SEM ±2.43); p<0.005; **Figure 3E**). Our findings here confirm earlier results that Huh-7.5 cells do not to secrete IL-1 β (Kofahi et al., 2016), even in the context of pyroptosis induction by LPS/Nigericin (**Figure 3E**). When THP-1 cells were co-cultured with HCV-infected Huh-7.5 cells, we found a significant increase in the amount of IL-1 β when compared to the uninfected co-culture (16.74 pmol/mL (SEM ±2.16) compared to 0.74 pmol/mL (SEM ±0.2), p<0.005). Since Huh-7.5 cells did not secrete IL-1 β, we can be confident that the changes in the levels of IL-1 β secretion in the co-culture can be attributed to pyroptosis induction in THP-1 cells. Together, these data suggest that THP-1 cells are susceptible to bystander pyroptosis when co-cultured with HCV-infected Huh-7.5 cells.

### 3.4 Bystander pyroptosis of Ramos and Jurkat cells co-cultured with HCV-infected Huh-7.5 cells

Given our findings that THP-1 cells undergo pyroptosis when co-cultured with HCV-infected Huh-7.5 cells, we were interested to determine whether other immune cell types were also susceptible to bystander pyroptosis in this context. To investigate this, either Ramos cells (B cell line) or Jurkat cells (T cell line) were co-cultured with HCV-infected or uninfected Huh-7.5 cells and caspase-1^+^ cells were quantified by flow cytometry. Caspase-1^+^ immune cells were quantified by gating on either CD19^+^ or CD3^+^ cells for Ramos or Jurkat cells, respectively. Interestingly, there was an increase in the total percentage of caspase-1^+^ cells, although not significant, when Ramos cells were co-cultured with HCV-infected cells compared to those co-cultured with uninfected Huh-7.5 cells (15.51% (SEM ±3.79) compared to 7.24% (SEM ±1.67), p=0.12; **Figure 4A**). However, when we focused on only the CD19^+^ cells from the co-culture, we did find a significant increase in the percentage of caspase-1^+^ Ramos cells when they were co-cultured with HCV-infected Huh-7.5 cells compared to the Ramos cells co-cultured with uninfected Huh-7.5 cells (3.75% (SEM ±0.44) compared to 1.07% (SEM ±0.35), p<0.05; **Figure 4A**). When we performed the same experiment with Jurkat cells, we found no significant differences in the percentage of caspase-1^+^ cells when Jurkat cells were exposed to HCV, when they were co-cultured with uninfected or infected Huh-7.5 cells, or when we measured the percentage of cells that were caspase-1^+^CD3^+^ (**Figure 4B**). The same experimental conditions were also used to evaluate IL-1 β release by ELISA, which showed no significant increase in secretion of IL-1 β in any of the experimental conditions for either Ramos or Jurkat mono- or co-cultures. We were unable to detect any IL-1 β secretion by Ramos cells, even from those treated with LPS/Nigericin, suggesting that Ramos cells do not produce IL-1 β, or it was produced at such low levels that it was undetectable by the assay. A very low amount of IL-1 β secretion was detected from Jurkat cells, suggesting that they do produce low amounts of IL-1 β. However, these amounts were not significantly different between untreated cells and those stimulated with LPS/Nigericin (2.06 pmol/mL (SEM ±0.6) compared to 2.82 pmoL/mL (SEM ±0.14); p=0.28; **Figure 4C**). IL-1 β secretion by THP-1 cells (previously included in **Figure 3E**) is included here for comparison purposes, as THP-1 cells secrete large quantities of IL-1 β, especially when stimulated with LPS/Nigericin. Together, these data suggest that there may be an effect of bystander pyroptosis when Ramos cells are co-cultured with HCV-infected Huh-7.5 cells, but future work should investigate this further in primary immune cells.

## 4 Discussion

Despite the availability of curative therapies for individuals living with HCV and decades of research attempting to understand HCV-induced pathogenesis, the exact mechanism by which HCV causes disease, particularly in individuals post-HCV cure, has yet to be fully understood. Previous work from our lab and others has suggested that programmed cell death, specifically pyroptosis, is involved in the pathogenesis associated with HCV infection (Negash et al., 2013; Shrivastava et al., 2013; Chattergoon et al., 2014; Kofahi et al., 2016; Negash et al., 2019; Wallace et al., 2022).

Research from our lab has previously documented the phenomenon of bystander pyroptosis in uninfected hepatocyte-like cells (Kofahi et al., 2016). The current study was conducted with the aim to further understand bystander pyroptosis induction by HCV *in vitro*.

This study set out to determine what triggers pyroptosis induction by HCV, and through this work we determined what steps of the HCV lifecycle could be the trigger that initiates HCV-induced pyroptosis. We found that neither UV- nor heat-inactivated HCV was sufficient to trigger pyroptosis in either Huh-7.5 or S29 cells, contrary to some earlier studies that suggested that binding of the viral glycoproteins with cell surface receptors could activate cell death (Nieto-Torres et al., 2015; Eisfeld et al., 2021; Lien et al., 2021a, 2021b). Inoculation of S29 cells with HCV was also insufficient to trigger pyroptosis. Together these findings suggest that HCV-induced pyroptosis is independent of entry/interactions with HCV virions at the cell surface (**Figure 1**).

To further dissect which step of the HCV life cycle could be involved in triggering pyroptosis, we transfected cells with various RNA constructs. By transfecting viral RNA (known as JFH1_T_ throughout this manuscript), we eliminate the effect of entry, and by using the S29 cell transfection model, we also remove the majority of the effect of infection of nearby cells by infectious virions. If left for an extended period of time, the cells transfected with JFH1_T_ RNA would eventually show significantly increased activation of caspase-1, but this would occur with multiple rounds of viral replication, just as it does if the cells are infected as opposed to transfected due to higher infection versus transfection efficiency. The ΔGDD construct, which renders the RdRp non-functional, confirmed that HCV RNA alone was unable to trigger pyroptosis. The JFH-sgr RNA encodes only the viral non-structural proteins. Although not significant when transfected into the Huh-7.5 cells, there was a significant increase in activated caspase-1 in S29 cells transfected with JFH-sgr. This suggests that in infected Huh-7.5 cells, fully infectious virus life cycle is required to trigger pyroptosis. The lack of pyroptosis induction by the JFH-sgr may be explained, in part, by the absence of p7 in this construct. Viroporins have been suggested to be a major factor in the induction of virus-induced inflammasome activation and pyroptosis (Ichinohe et al., 2010; Triantafilou et al., 2013; Chen et al., 2019), and p7 of HCV has been suggested to act as a viroporin in a similar manner (Farag et al., 2017; Breitinger et al., 2021). However, in uninfected S29 cells, merely exposure to HCV or production of non-structural proteins was sufficient to trigger pyroptosis (**Figure 2**). The reason as to why transfection of the JFH-sgr RNA into S29 cells, but not Huh-7.5 cells, was sufficient to trigger pyroptosis is likely because S29 cells have retained threonine at position 55 of the RIG-I gene (**Figure 2F**), allowing for functional RIG-I detection of double-stranded RNA in the cytoplasm (Sumpter et al., 2005).

Early studies on pyroptosis induction by HCV were done to follow up on findings of elevated IL-1 β and IL-18 in serum of individuals infected with HCV (Spanakis et al., 2002; Falasca et al., 2006; Chattergoon et al., 2011; Veenhuis et al., 2016). Knowing that hepatocytes are not particularly inflammatory in nature, many researchers wondered whether monocytes or macrophages might be the cause of these elevated cytokine levels in serum. THP-1 cells were used for some early studies that examined whether these cells could be infected with HCV or whether viral RNA was sufficient to trigger pyroptosis in these cells. However, to our knowledge, there has been no research until now on these cells through a lens of bystander pyroptosis. Our findings that THP-1 cells undergo pyroptosis during co-culture with HCV-infected Huh-7.5 cells (**Figure 3**) suggest that this could be an explanation for the documented instances of elevated pyroptosis-associated cytokines in the sera of HCV-infected individuals. It may also help to explain why the levels of these cytokines remain elevated even following DAA cure (Burchill et al., 2017). If the factor that triggers pyroptosis of these cells is not directly related to HCV, it is conceivable that the factor that does trigger pyroptosis of these cells is not eliminated despite the elimination of virus. However, this warrants additional investigation and should be the focus of future studies.

Interestingly, a few years ago, it was shown that abortively infected CD4^+^ T cells die by pyroptosis in a bystander-like manner, and this is thought to contribute to the pathogenesis and loss of CD4^+^ T cells associated with HIV infection (Doitsh et al., 2014; Monroe et al., 2014; Zhang et al., 2021). Although the effect was small and may not be relevant *in vivo*, our data suggests that B cells may undergo bystander pyroptosis whereas T cells do not (**Figure 4**). This is not completely surprising, as HCV is rarely reported in conjunction with lymphopenia. Regardless, this is interesting information to consider, as it suggests that although the adaptive immune cells may be dysfunctional during chronic HCV infection, these cells do not appear to be dying, at least not by pyroptosis, in our system. Future work should expand this to look at other innate immune cell types, as well as the use of PBMCs.

Our *in vitro* system is inherently limited in its utility of assessing whether any of our reported findings occur *in vivo*. Our study made use of a highly infectious, although cell culture-adapted, strain of HCV, JFH1_T_, and Huh-7.5 cells, which are human hepatoma-derived. It is possible that that inflammasome activation differs between primary hepatocytes and our cell lines, which could limit the generalizability of the results presented here. Future work would benefit from performing similar studies in primary cells. The system we used here has many advantages such as its utility for identifying drug targets and its ability to replicate HCV at high titres (reviewed by Tellinghuisen et al., 2007), however, it also has limitations, including the fact that Huh-7.5 cells have been reported to have deficiencies in certain innate immune pathways (Sumpter et al., 2005; Kawamoto et al., 2020). JFH1_T_ is also unique as it is the only patient isolate of HCV that is known to have grown in cell culture without adaptive mutations (Kato et al., 2003). Inherent limitations associated with cell culture models should also be considered, and any findings presented here should be followed up by using animal models and/or patient biopsies.

Despite the limitations of our *in vitro* approach, our findings serve as motivation for follow-up *in vivo* research. Our study cannot definitively say that fully infectious life cycles are necessary for pyroptosis induction, or that monocytes/macrophages undergo bystander pyroptosis within the liver of an HCV-infected individual. However, other literature supports our findings by reporting signs of pyroptosis in individuals living with HCV (Falasca et al., 2006; Chattergoon et al., 2011; Veenhuis et al., 2016; Burchill et al., 2017). It is also important to consider these findings given the fact that more recent studies have documented ongoing inflammation and liver disease even in individuals who were cured of HCV using DAAs, suggesting ongoing inflammation even in the absence of productive infection (Welsch et al., 2017; Abe et al., 2020; D’Ambrosio et al., 2021; Montaldo et al., 2021). Other viruses have also been reported to activate the inflammasome/pyroptosis pathway *in vivo*, as have been reported with SARS-CoV-2 (Courjon et al., 2021; Rodrigues et al., 2021; Sefik et al., 2022; Peleman et al., 2023), influenza A virus (Bauer et al., 2012), HBV (Falasca et al., 2006; Han et al., 2015; Askari et al., 2016; Chen et al., 2018), and HIV (Veenhuis et al., 2016; Ahmad et al., 2018; Zhang et al., 2021). Importantly, future studies should address whether inflammatory markers remain elevated among individuals even following HCV cure (as suggested by Burchill et al., 2017), and in liver biopsies from individuals with ongoing complications post-cure.

## 5 Conclusions

In this study, we determined that infectious HCV virion production is necessary for substantial pyroptosis induction and that surface interactions of the viral glycoproteins with cell surface receptors, intracellular viral RNA alone, or production of the non-structural proteins were insufficient to trigger pyroptosis in isolation in Huh-7.5 cells. We also provide additional support to previous literature that suggests monocytes are impacted by pyroptosis induction in the context of HCV (i.e. bystander pyroptosis). However, this pattern does not appear to be observed in the same capacity in the case of B or T cell lines. Overall, these findings add to the overall understanding of the mechanism of HCV-induced pyroptosis, support previously published data suggesting that uninfected THP-1 cells can undergo pyroptosis, and show that B and T cells do not appear to be affected by bystander pyroptosis in the context of HCV infection.

## Data Availability

The raw data supporting the conclusions of this article will be made available by the authors, without undue reservation.
