# Supplementary material for: The inflammasome next door: characterizing pyroptosis induction in HCV-infected and uninfected bystander cells in vitro
Source: Front Cell Infect Microbiol. 2026 Feb 3;15:1603739. doi: 10.3389/fcimb.2025.1603739 (PMC12960644; doi:10.3389/fcimb.2025.1603739)
Supplement: Supplementary file 1 [file DataSheet1.pdf]

Supplementary Materials

The Inflammasome Next Door: Characterizing Pyroptosis Induction in HCV-infected and Uninfected Bystander Cells *in vitro*

Hannah L. Wallace[1], Cassandra L. Gardner[1], Calvin N. Ezeanyaegbu[1], Jordan Wight[2], Andrew S. Lang[2], and Rodney S. Russell[1]

[1]Immunology and Infectious Diseases, Division of Biomedical Sciences, Faculty of Medicine, Memorial University, St John's, NL, Canada, [2]Department of Biology, Faculty of Science, Memorial University, St John's, NL, Canada

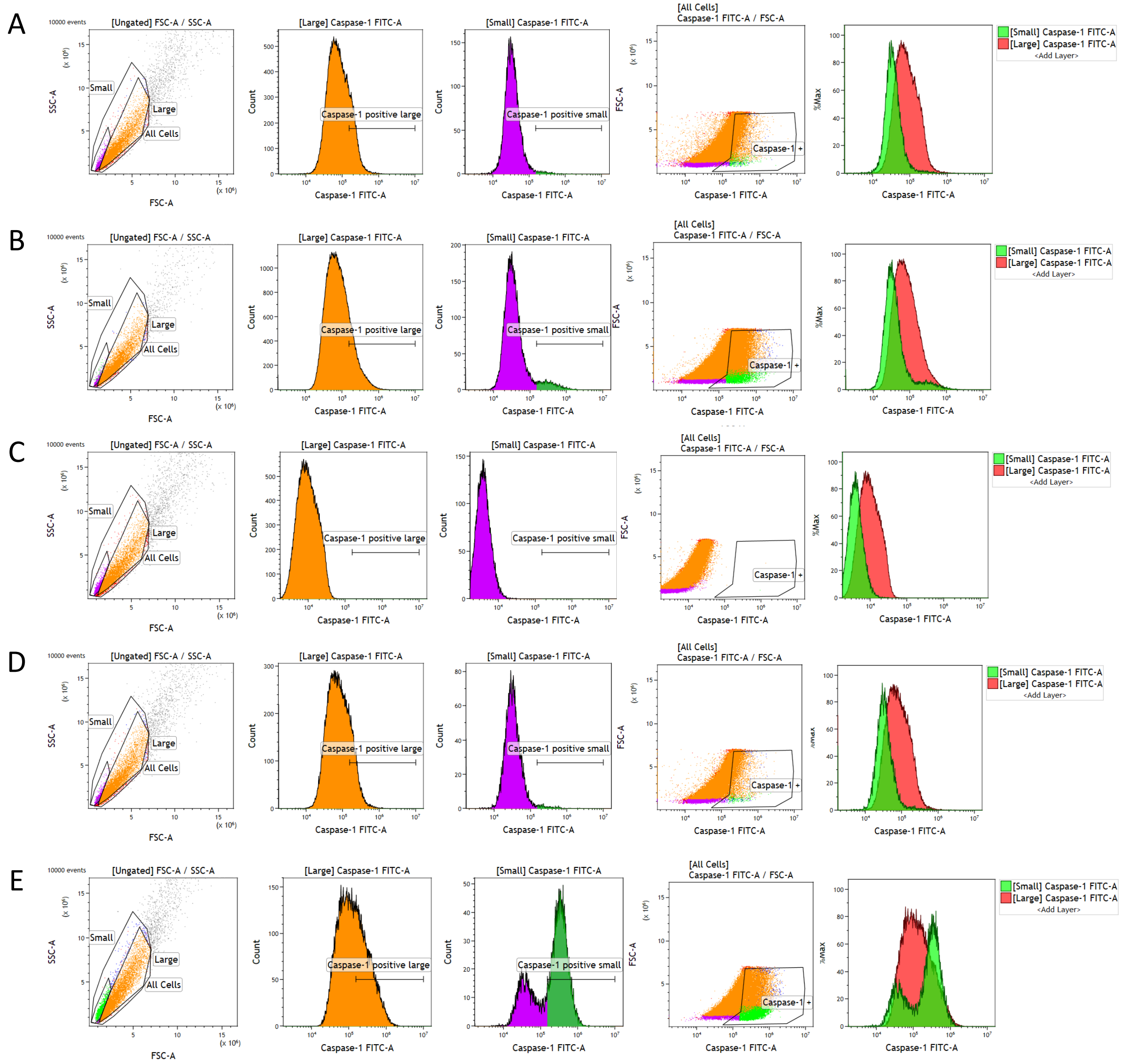

Supplementary Figure 1. Flow cytometry gating strategy. All gating strategies were based on Huh-7.5 cells, for which representative examples are shown here. This is due to the heterogeneous nature of the Huh-7.5 cell population in comparison to the immune cell populations. Forward versus side scatter plots were used to gate cell populations. Histograms were used to gate caspase-1 positivity (caspase-1+) for each cell population of interest. Forward scatter versus caspase-1 probe fluorescence intensity was also used to confirm gated positivity. (**A**) Huh-7.5 cells, heat-shocked, caspase-1 stained. (**B**) Huh-7.5 cells, LPS/Nigericin-treated, caspase-1 stained. (**C**) Huh-7.5 cells, uninfected, unstained. (**D**) Huh-7.5 cells, uninfected, caspase-1 stained. (**E**) Huh-7.5 cells HCV-infected, caspase-1 stained. (**A–E**) For each condition, a minimum of 10,000 events were collected. For experimental conditions, 10,000 events were collected in the caspase-1+ population, meaning that far

more than 10,000 cells were collected in total. For detailed explanation of the caspase-1 gating strategy see Wallace et al., 2022.

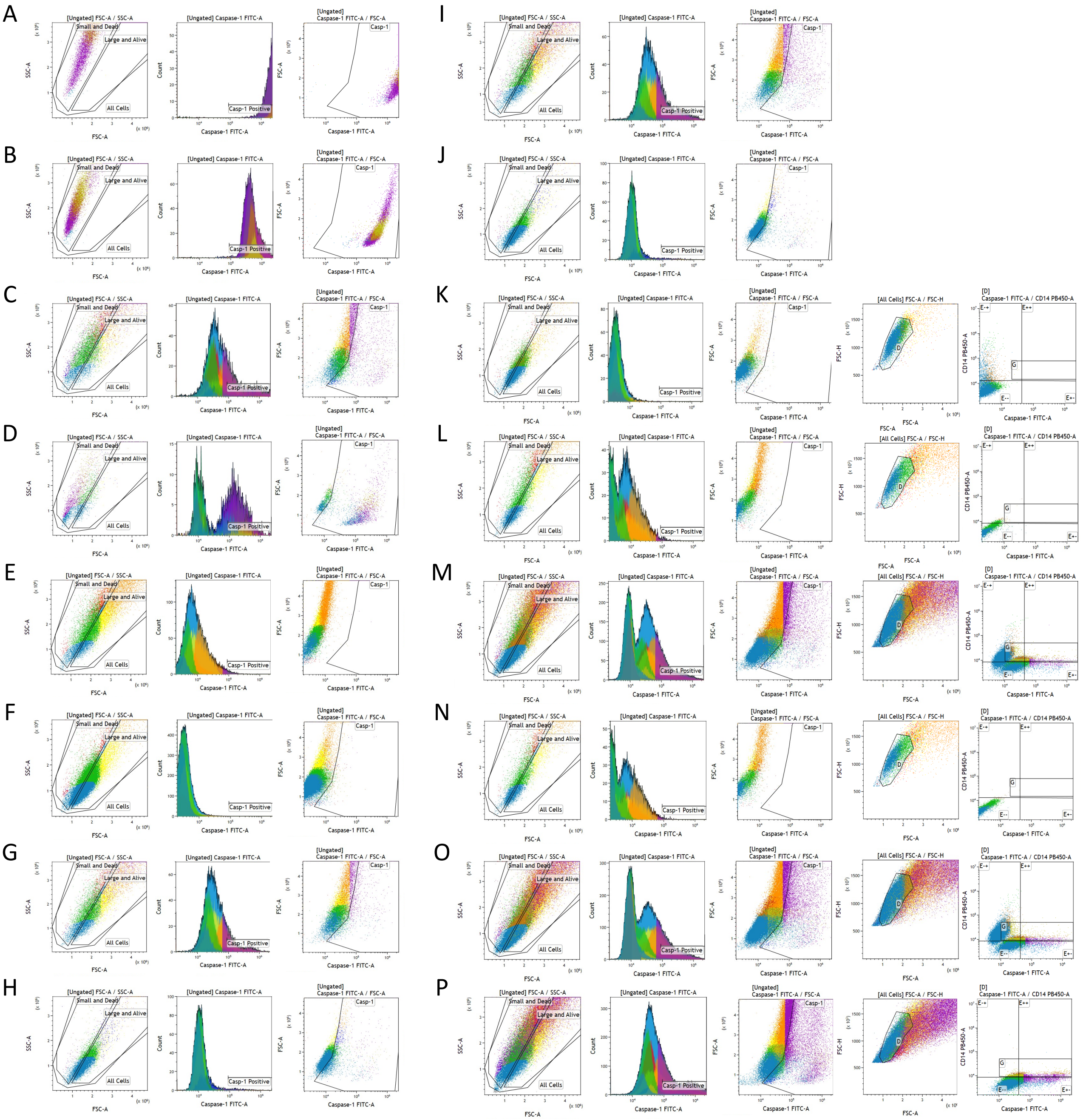

Supplementary Figure 2. Flow cytometry gating strategy for immune cell co-culture experiments. All gating strategies were based on Huh-7.5 cells, for which representative examples are shown here. Forward versus side scatter plots were used to gate cell populations. Histograms were used to gate caspase-1 positivity (caspase-1+) for each cell population of interest. Forward scatter versus caspase-1 probe fluorescence intensity was also used to confirm gated positivity. (**A**) Huh-7.5 cells, heat shock, caspase-1 stained. (**B**) THP-1 cells, heat shock, caspase-1 stained. (**C**) Huh-7.5 cells, LPS/Nigericin-treated, caspase-1 stained. (**D**) THP-1 cells, LPS/Nigericin-treated, caspase-1 stained. (**E**) Huh-7.5 cells, uninfected, unstained. (**F**) THP-1 cells, untreated, unstained. (**G**) Huh-7.5 cells, uninfected, caspase-1 stained. (**H**) THP-1 cells, untreated, caspase-1 stained. (**I**) Huh-7.5 cells, HCV-infected, caspase-1 stained. (**J–P**) Forward scatter height versus forward scatter area was used to gate

on smaller cells (includes all immune cells and smaller Huh-7.5 cells; gate D). CD14 fluorescence intensity versus caspase-1 probe fluorescence intensity was used to gate on double positive cells. (**J**) THP-1 cells, 'HCV infected,' caspase-1 stained. (**K**) THP-1 cells, untreated, CD14 stained. (**L**) Huh-7.5 and THP-1 co-culture, uninfected, unstained. (**M**) Huh-7.5 and THP-1 co-culture, uninfected, Caspase-1 and CD14 stained. (**N**) Huh-7.5 and THP-1 co-culture, HCV-infected, unstained. (**O**) Huh-7.5 and THP-1 co-culture, HCV-infected, caspase-1 and CD14 stained. (**P**) Huh-7.5 and THP-1 coculture, LPS/nigericin-treated, caspase-1 and CD14 stained.

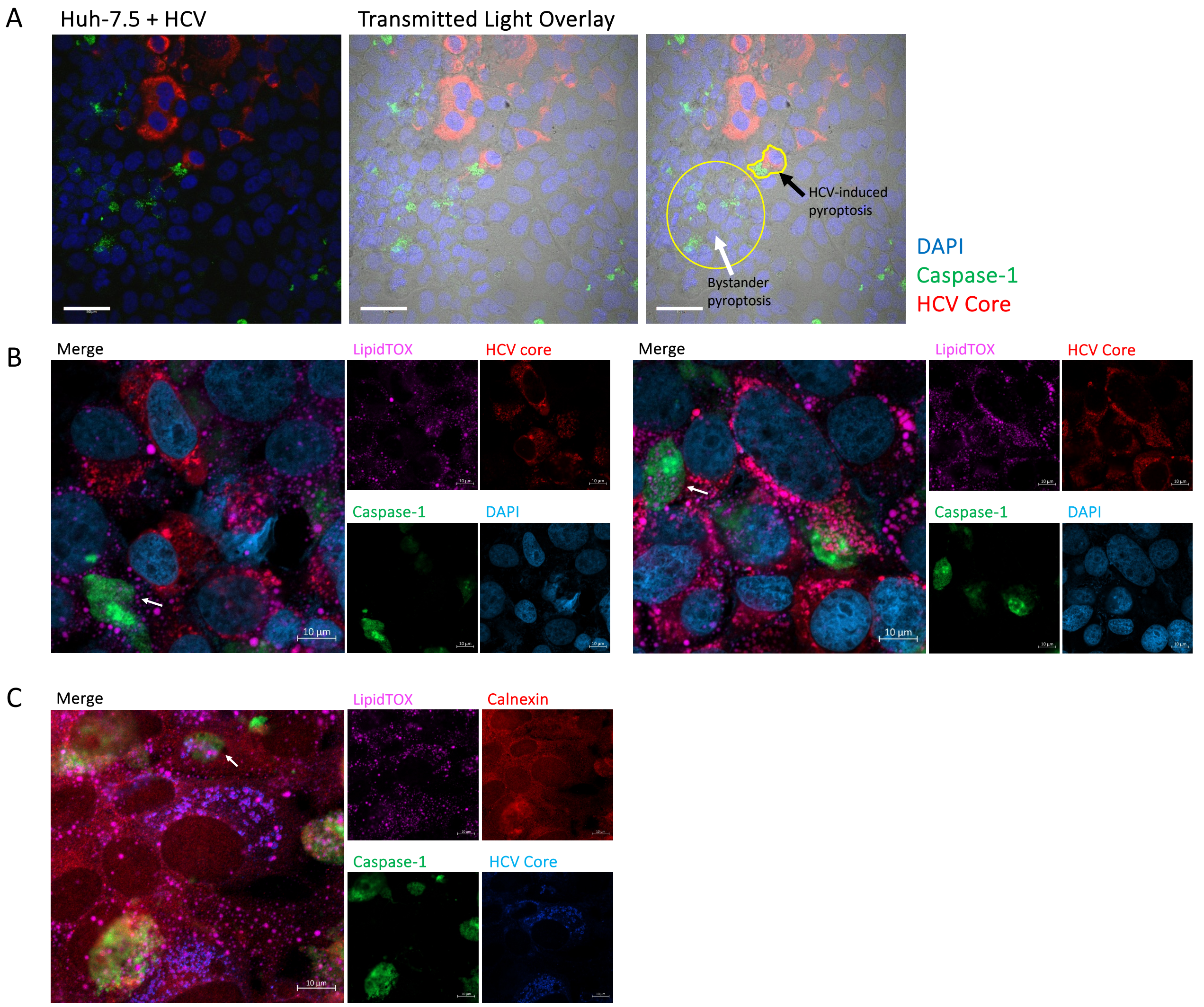

Supplementary Figure 3. HCV triggers pyroptosis in infected, as well as uninfected, bystander Huh-7.5 cells. Huh-7.5 cells were infected with HCV at MOI = 1 or left uninfected. At 3 dpi, cells were fixed and stained for (**A**) cleaved caspase-1 (green), HCV core (red) or (**B**) cleaved caspase-1 (green), HCV core (red), lipid droplets (pink) and (**A, B**) nuclei were stained using DAPI (blue), or (**C**) cleaved caspase-1 (green), HCV core (blue), lipid droplets (pink), and calnexin (red). (**A–C**) Analysis was performed using fluorescence microscopy. (**A**) Transmitted light overlay and labeled copy of the image included. Black arrow indicates an HCV-infected cell undergoing pyroptosis. (**B**) Two representative images. (**A–C**) White arrow indicates a cell undergoing bystander pyroptosis. (**A**) Scale bar, 100 μm. (**B, C**) Scale bar, 10 μm. (**A–C**) Data are representative of at least three independent experiments.

Supplementary Table 1. Primers used for amplification of the RIG-I transcript. RIG-I_F and RIG-I_R were designed based on the 5' and 3' ends of the mRNA transcript (GenBank NM_0143114.4), while all other primers were designed based on the complete coding sequence (GenBank NG_046918.1), with the positions of the first base of these primers indicated in the primer name.

Fragment	Primer Name	Primer Sequence (5'->3')	Amplicon size (bp)
Fragment 1	RIG-I_F	AACGTAGCTAGCTGCAAGC	522
	RIG-I_493R	CCTTGTCTGATCTGAGAAG	
Fragment 2	RIG-I_F	AACGTAGCTAGCTGCAAGC	938
	RIG-I_909R	ATACACTGGGATCTGATTCG	
Fragment 3	RIG-I_401F	AGGAATGTGAAGAAATTCTAC	1038
	RIG-I_1438R	TCATCAGCTGAGCTATGATG	
Fragment 4	RIG-I_820F	TCACTGCTTATATGTGAACATC	1171
	RIG-I_1990R	GTCCAGTCAATATGCCAG	
Fragment 5	RIG-I_1303F	GATGCGTCAGTGATAGCAAC	1159
	RIG-I_2461R	CTGTGTAACATGCCAAGG	
Fragment 6	RIG-I_1847F	TCATCTTACAAGAAGAGTACC	1009
	RIG-I_R	TGATTATACCCACTATGTTTG	
Fragment 7	RIG-I_2337F	GATTCTGCATATACAGACTC	519
	RIG-I_R	TGATTATACCCACTATGTTTG	

Supplementary Table 2. The mean C_T of viral RNA from Huh-7.5 and S29 cells following transfection with JFH-sgr (sub-genomic replicon) at various times post-transfection.

Time Post-transfection	Huh-7.5 Mean C_T	S29 Mean C_T
1 hour	9.564	8.780
1 day	10.459	9.864
2 days	9.935	10.768
3 days	10.801	12.866
4 days	11.512	12.058
